# Design of a Flexible Wasp-Inspired Tissue Transport Mechanism

**DOI:** 10.3389/fbioe.2021.782037

**Published:** 2021-11-09

**Authors:** Esther P. de Kater, Aimée Sakes, Jette Bloemberg, David J. Jager, Paul Breedveld

**Affiliations:** ^1^ Department of BioMechanical Engineering, Bio-Inspired Technology Group, Faculty of Mechanical, Maritime, and Materials Engineering, Delft University of Technology, Delft, Netherlands; ^2^ Department of Electronic and Mechanical Support Division, Faculty of Electrical Engineering, Mathematics and Computer Science, Delft University of Technology, Delft, Netherlands

**Keywords:** medical device design, tissue transportation, minimally invasive surgery, biomimetic, bio-inspired design

## Abstract

Tissue transport is a challenge during Minimally Invasive Surgery (MIS) with the current suction-based instruments as the increasing length and miniaturisation of the outer diameter requires a higher pressure. Inspired by the wasp ovipositor, a slender and bendable organ through which eggs can be transported, a flexible transport mechanism for tissue was developed that does not require a pressure gradient. The flexible shaft of the mechanism consists of ring magnets and cables that can translate in a similar manner as the valves in the wasp ovipositor. The designed transport mechanism was able to transport 10wt% gelatine tissue phantoms with the shaft in straight and curved positions and in vertical orientation against gravity. The transport rate can be increased by increasing the rotational velocity of the cam. A rotational velocity of 25 RPM resulted in a transport rate of 0.8 mm/s and increasing the rotation velocity of the cam to 80 RPM increased the transport rate to 2.3 mm/s though the stroke efficiency decreased by increasing the rotational velocity of the cam. The transport performance of the flexible transport mechanism is promising. This means of transportation could in the future be an alternative for tissue transport during MIS.

## Introduction

### Tissue Transportation During Surgery

The transportation of gasses, liquids and solids in the human body is a key function that needs to be fulfilled to support life. Disturbances to these processes can cause serious symptoms and often require surgical treatment to resolve the issue. Treatment often involves the removal and, therefore, transportation, of the disturbance, such as thrombus, cancerous or infectious tissue, from the intervention site to outside the patient’s body (Spear, 2010; Meissner, 2012). The removal, and thus transportation, of liquids and solids from the body is also of vital importance for diagnostic purposes (Hong et al., 2013). Vice versa, the transportation of substances, such as medicine (Chin et al., 2005) or radioactive particles (Kwon et al., 1991), from outside the patients’ body towards a target area inside the body is essential for treatment purposes.

Whereas the removal of tissues is generally a relatively straightforward procedure in open surgery, it is more challenging in Minimally Invasive Surgery (MIS). During open surgery a relatively large incision is made, allowing for easier removal of the obstruction, as the distance between the surgical opening and the intervention site is short and the path is wide. Nowadays, more and more surgeries are performed minimally invasive, in which one or multiple small (5–15 mm) incisions are made through which entry to the body is obtained. MIS is associated with positive primary and secondary outcomes, such as shorter hospital stays and less postoperative pain (Jaschinski et al., 2015). One major downside of MIS is, however, that the transportation and subsequent removal of tissues is more challenging due to the small incision size and a longer, sometimes curved, pathway between the intervention site and the incision.

### Suction-Based Instruments

Suction-based instruments are the current standard in MIS to transport a variety of substances from the operation area to outside the patient’s body. Transportation is achieved by creating a pressure gradient in a tubular structure, such that substances in front of the tube tip are sucked in and can, subsequently, be removed.

**FIGURE 1:**
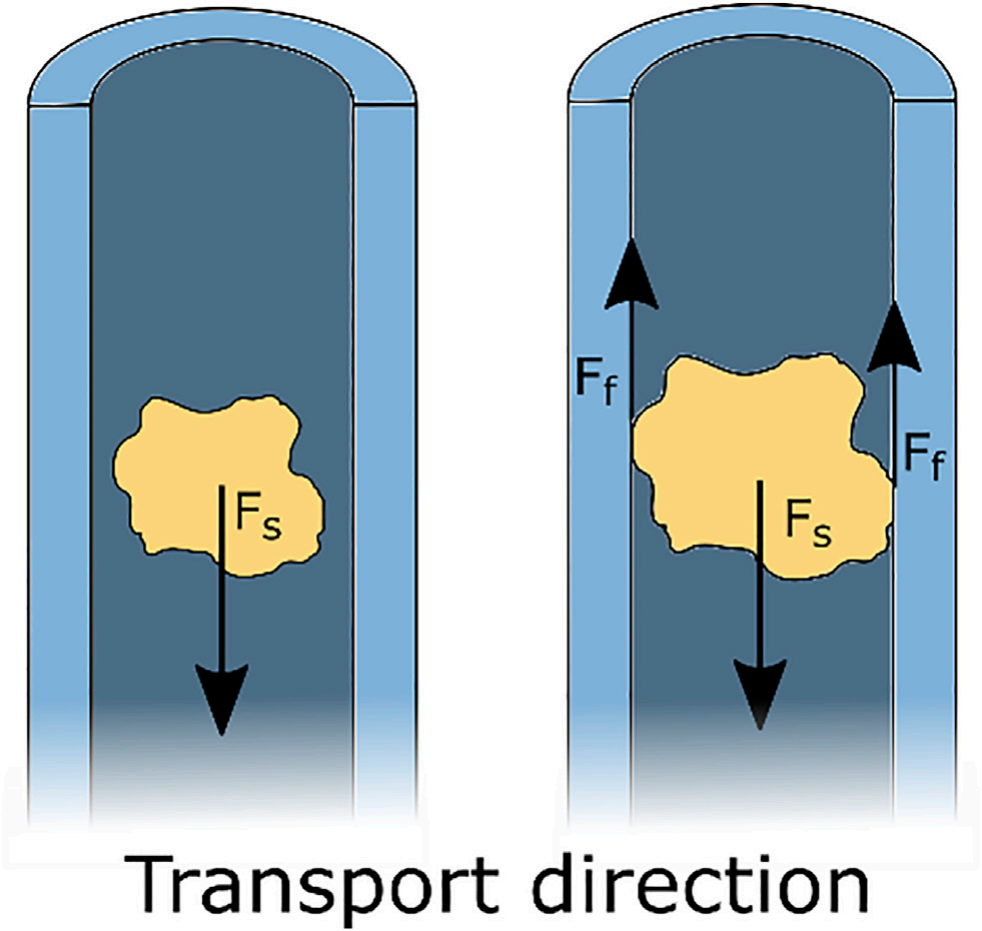
Schematic representation of suction-based transport. A suction force *F*_*s*_ acts on the tissue, resulting in transportation of the tissue. Clogging occurs when the piece of tissue is in contact with the walls of the tube and the resulting friction forces $F_{f}\;$ are larger than the suction force $F_{s}$ . Clogging prevents any further transportation of the tissue part.

Although suction-based instruments function generally well, they become less effective with the ongoing trend of miniaturisation. Restrictions to the outer dimensions pose a problem for suction-based instruments, as a smaller lumen diameter and a longer tube require a higher pressure difference to achieve adequate transportation (Hu and Stiefel, 2016). Another often occurring problem during suction-based transport is clogging. Clogging is mainly the result of friction between the transported tissue and the instrument’s wall (Rioufol et al., 2006), see Figure 1. Clogging makes further transportation impossible and requires removal of the clogged tissue part, with an increase in the procedure time as a result. Therefore, a reliable alternative for suction-based transport that is not prone to clogging is needed.

### Biological Inspiration: Wasp Ovipositor

**FIGURE 2:**
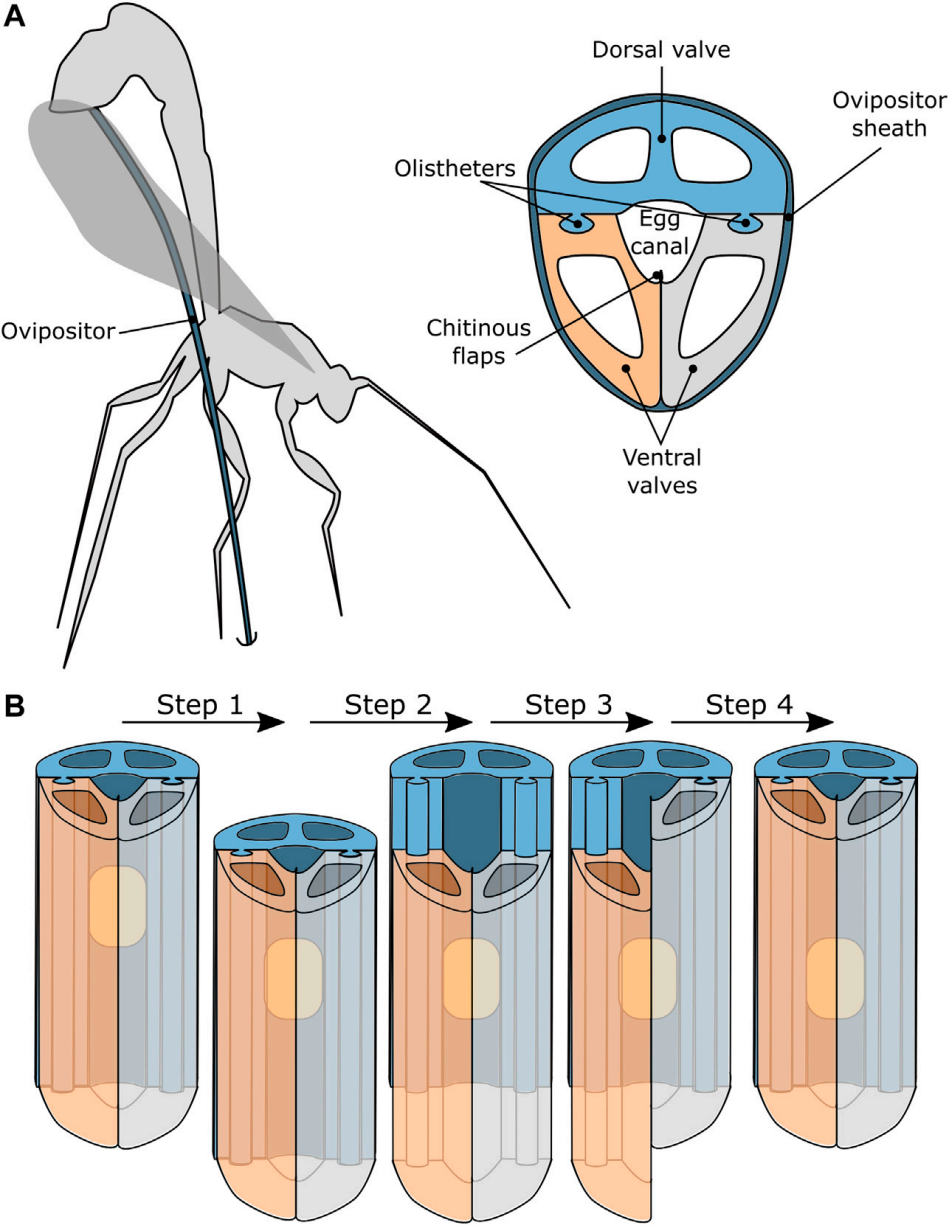
Schematic representation of a parasitic wasp that uses a needle-like organ called an ovipositor to transport eggs into host material (Austin and Browning, 1981; Cerkvenik et al., 2017; van Meer et al., 2020). **(A)** The ovipositor generally consists of one dorsal valve and two ventral valves that are held together by tongue-and-groove connections, called olistheters, that allow the valves to slide with respect to each other. The chitinous flaps prevent the egg from escaping the egg channel and the ovipositor is covered by the ovipositor sheath. **(B)** A possible motion sequence of the valves that results in transport of the eggs along the ovipositor.

An interesting biological example of reliable transport through a very slender tube can be found in parasitic wasps. Parasitic wasps possess an ovipositor: a thin and flexible tubular organ with which they can drill in a living host, to deposit their eggs inside this host, see Figure 2A. The egg transport through the ovipositor is not achieved by a pressure gradient, such as in suction-based instruments, but by an oscillatory motion of the ovipositor valves that together form the tubular ovipositor (Austin and Browning, 1981; Cerkvenik et al., 2017; van Meer et al., 2020).

The ovipositor generally consists of three independently translating valves: one dorsal valve and two ventral valves. The valves are enclosed by the ovipositor sheath and can slide axially with respect to each other while being kept in place radially by a tongue-and-groove connection, called olistheter (Ahmed et al., 2013). The chitinous flaps of the ventral valves are thought to provide a seal that prevents the egg from escaping the egg channel during oviposition (Ahmed et al., 2013).

It is hypothesised that the egg transport relies on the friction between the egg and the valves (Austin and Browning, 1981; Ahmed et al., 2013). The exact motion sequence that results in the transportation of the eggs is, however, not fully known. One hypothesis is that the transportation of the egg and the drilling occurs simultaneously. The sequence of valve movements would result in drilling the ovipositor deeper into the substrate and simultaneously transport and deposition of the eggs. A second hypothesis is that the wasp can transport eggs through the ovipositor without drilling the ovipositor deeper into the substrate. In this scenario, the transportation is thought to be achieved by small translations of the ovipositor valves in a repeating sequence, see Figure 2B.

Following the second hypothesis, the egg transport sequence starts with all three valves translating in the desired transport direction, see Figure 2B Step 1. The egg will move along with the valves due to the friction between the egg and the valves. Subsequently one of the valves retracts while the other two valves remain stationary, see Figure 2B Step 2. It is important to note that the wasp’s eggs are somewhat flexible resulting in the friction force being dependent on the contact surface between the egg and the valve. Therefore, the egg will remain stationary, as the combined friction with the two stationary valves exceeds the friction with the single retracting valve, assuming that all valves have the same contact surface with the egg. After the retraction of the first valve, the second valve retracts, while the other two valves remain stationary, see Figure 2B Step 3. Again, the egg will remain stationary. The final step in the sequence is the retraction of the third valve while the others remain stationary, see Figure 2B Step 4. After this step, all the valves are back in their initial position, while the egg has moved one stroke length in the transport direction.

This cycle is repeated until the egg has been transported along the entire length of the ovipositor. This transport method is called friction-based transport, as the friction between the egg and the valve is what effectuates the transportation.

### Friction-Based Transport

**FIGURE 3:**
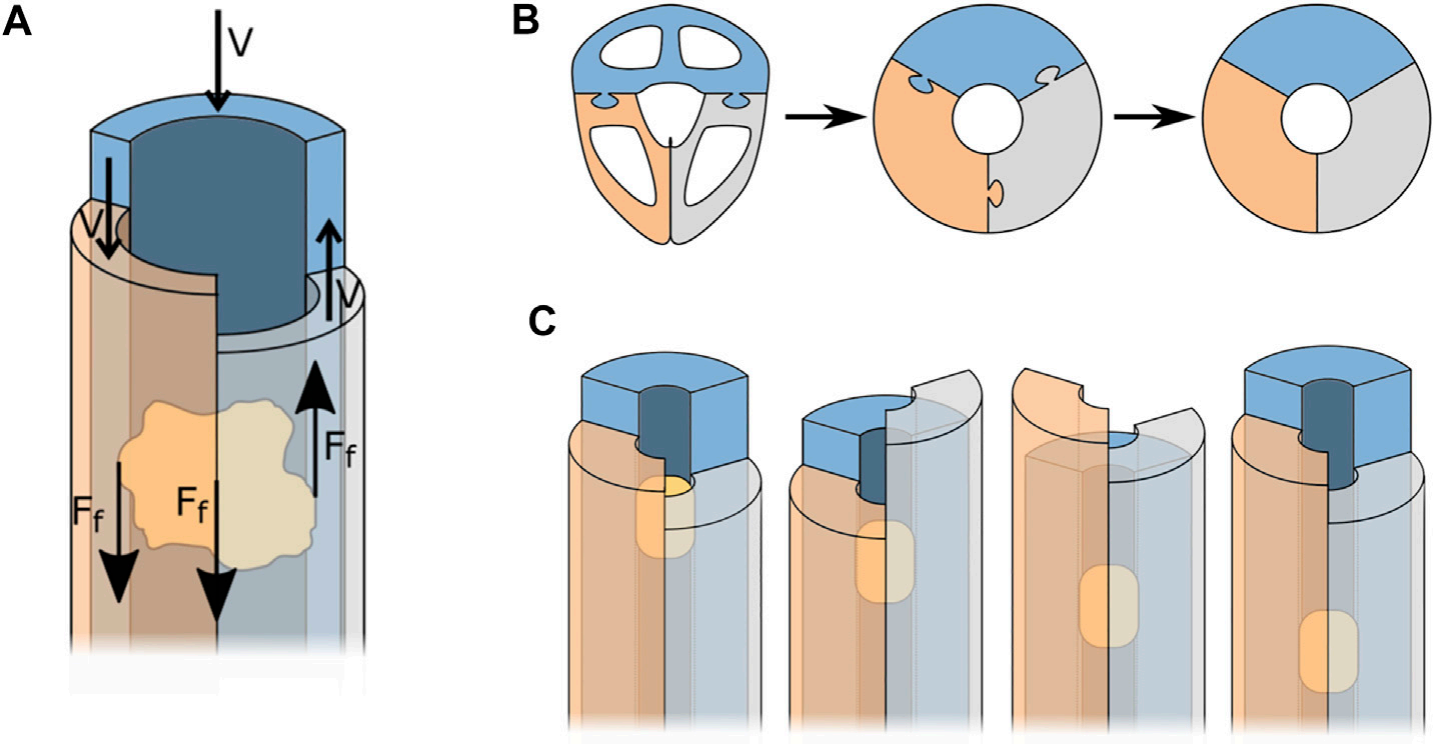
Simplification of the wasp ovipositor to three semi-circular blades that form a tube. **(A)** Forces acting during friction-based transport. **(B)** Simplification process of the ovipositor. **(C)** Friction-based transport, for explanation see text.

In suction-based instruments, the friction between the transported tissue and the walls of the instrument is a major cause of clogging. This problem does not occur in the wasp’s ovipositor, as the friction between the transported egg and the valves is what effectuates the transportation, see Figure 3A. Friction-based transport could, therefore, be an interesting alternative for suction-based transport.

To better understand how to apply the working principle of egg transport in the design of a tissue transport mechanism, the shape of the ovipositor was simplified as a cylinder held together by the olistheters. Further simplification of the ovipositor valves results in three semi-circular blades that form a circular lumen through which the tissue can be transported, see Figure 3B.

Transportation of the tissue is achieved once the resultant friction force on the tissue is larger in the transport direction than in the opposite direction. The resultant friction force ${\vec{F}}_{\text{friction}}$ [N] is the sum of the friction forces induced by each of the blades and can be written as illustrated in Eq. 1. Assuming that with relatively small pieces of tissue the effect of gravity can be neglected, that the tissue is flexible such that the generated friction is dependent on the contact surface, that the inner surface properties and material of the blades are identical, and that the tissue has full contact with each of the three blades, each blade results in the same absolute value of friction force, see Eq. 2. A possible motion sequence for the blades is depicted in Figure 3C. In this motion sequence, two valves advance in the transport direction, while one valve simultaneously retracts. This results in a resultant friction force in the transport direction that is twice as large as in the opposite direction, see Eq. 3. A system comprising of more than three blades will also be able to transport the tissue in the desired direction as long as Eq. 3 holds.

$${\vec{F}}_{\text{friction}}={\vec{F}}_{\text{friction}\;1}+{\vec{F}}_{\text{friction}\;2}+{\vec{F}}_{\text{friction}\;3}$$ (1)

$$\left|{\vec{F}}_{\text{friction}\;1}\right|=\left|{\vec{F}}_{\text{friction}\;2}\right|=\left|{\vec{F}}_{\text{friction}\;3}\right|$$ (2)

$$\sum {\vec{F}}_{\text{friction}\;\text{advancing}\;\text{valves}}>\sum {\vec{F}}_{\text{friction}\;\text{retracting}\;\text{valves}}$$ (3)

### Ovipositor Inspired Instruments

The ovipositor has been a source of inspiration for a number of designs. Multiple self-propelling needles have been developed (Frasson et al., 2010; Ko et al., 2010; Sprang et al., 2016), of which some are even steerable (Scali et al., 2017a; Scali et al., 2017b). Furthermore, friction-based transport has been used in the development of a rigid tissue transport mechanism described by Sakes et al. (2020). This mechanism can transport tissue phantoms with a variety of material characteristics without the need for a pressure differential. The instrument consists of six reciprocating semi-cylindrical blades that mimic the valves of the ovipositor, similar to the system shown in Figure 3C.

### Goal of This Study

Friction-based transport could be a viable alternative for the currently used suction-based tissue transport mechanisms, but has till now only been used in rigid form. The use of a flexible friction-based tissue transport mechanism increases the area that can be reached as the instrument can bend to follow the patient’s anatomy, which could be interesting for a wide variety of medical procedures, such as the removal of tissue in gastro-intestinal interventions, Ear Nose Throat (ENT) or endo-nasal skull base surgery, or in cardiovascular procedures, e.g., for the removal of occluded tissue from blood vessels. The goal of this study is, therefore, to explore a novel design for a flexible friction-based tissue transporter and to evaluate its use for future surgical applications.

## Proposed Design

### Flexible Valves

**FIGURE 4:**
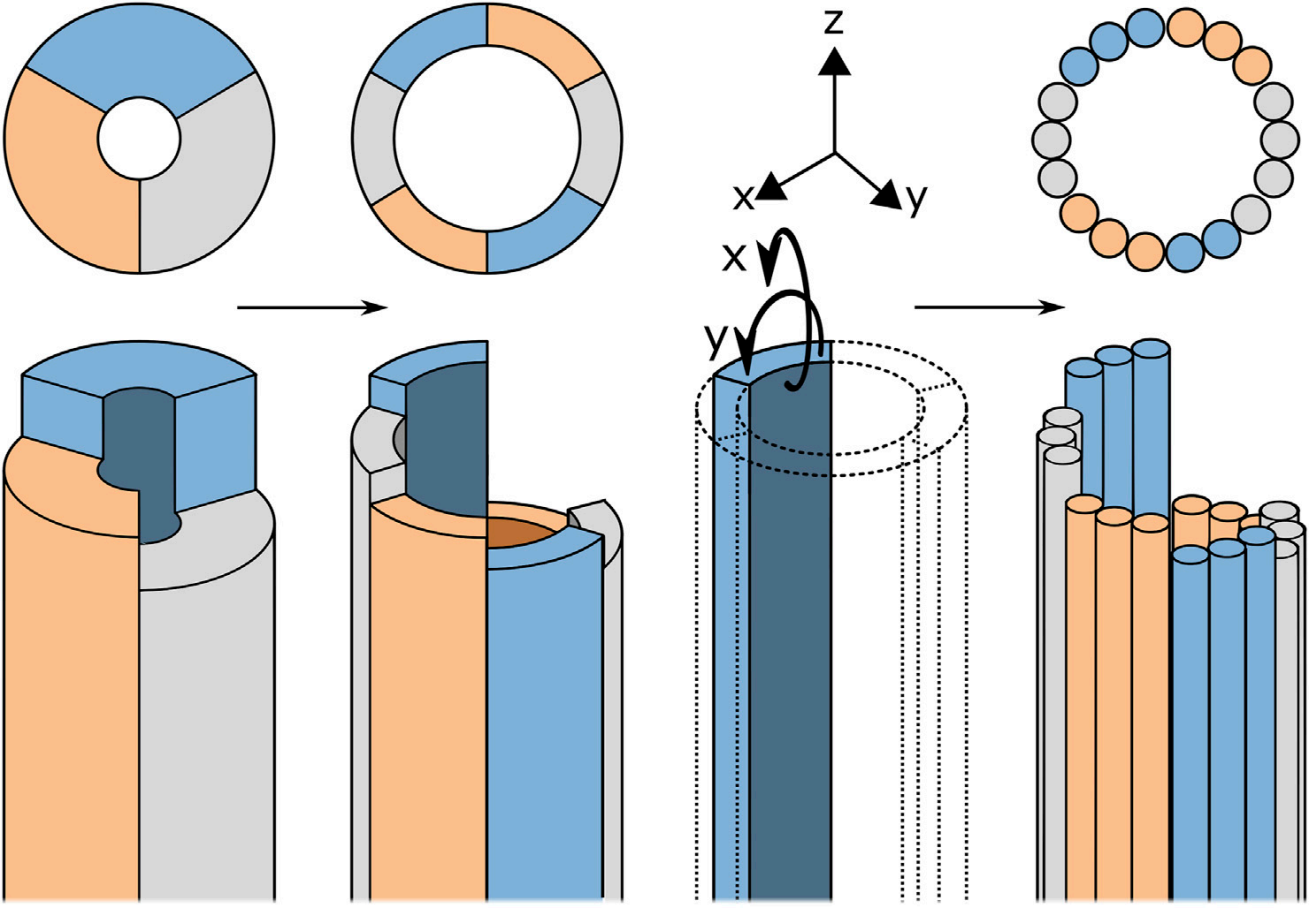
Schematic representation of the design process from the rigid transport mechanism described by Sakes et al. (2020)) to the flexible blades. For explanation, see text.

Following the ovipositor mechanism, the basis of the proposed design are valves that, through small oscillatory translations, transport the tissue. In the design of Sakes et al. (2020), the complex-shaped valves of the wasp are simplified to rigid semi-circular blades, see Figure 4. These semi-circular blades are rigid but could be made out of flexible material in order to create a flexible transport mechanism. However, semi-circular blades have a preferred axis of bending, allowing relatively easy bending around the x-axis, see in Figure 4, but a strong resistance to bending around the y-axis. Together, the blades form a tubular structure and bending this tube would always require multiple blades to bend in their unpreferred direction. Therefore, an alternative must be found for these semi-circular blades. Structures with a round cross-section do not have a preferred axis of bending, ensuring equal flexibility in both bending planes. It was therefore chosen to use cylindrical blades that exhibit high flexibility, such as cables.

### Lumen Formation

**FIGURE 5:**
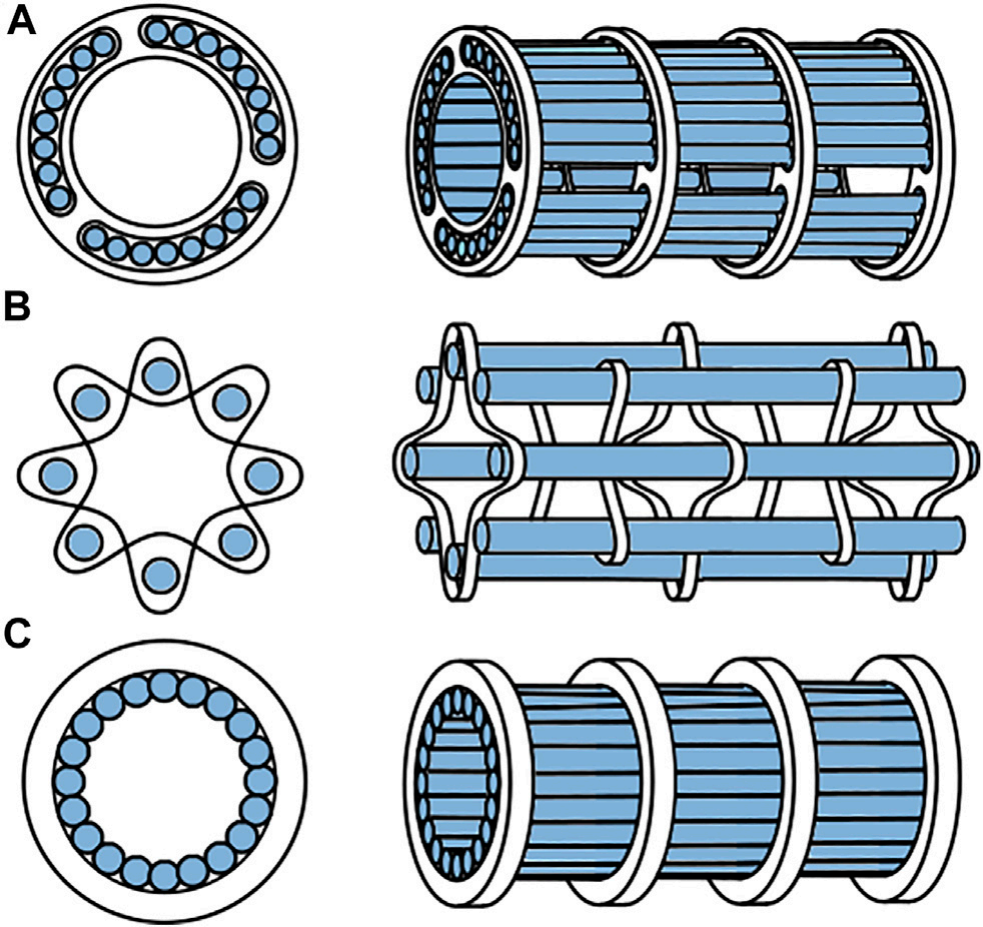
Different options to create an open lumen while still allowing the cables to translate. **(A)** rings with holes, **(B)** woven structures, **(C)** ring magnets.

The cables that mimic the ovipositor valves need to form a tubular structure with a constant lumen diameter through which the tissue can be transported. It was decided to create the shaft using eighteen 0.6 mm cables such that the tubular structure would have an inner diameter of approximately 3.8 mm. The tongue-and-groove interlocking mechanism in wasp ovipositors is nearly impossible to manufacture in combination with cables. A number of alternative solutions that prevent radial movement of the cables while allowing the cables to translate in the longitudinal direction are presented in Figure 5. To ensure uninterrupted contact between the cables and the to be transported tissues, the solution should not contain elements that protrude inside the lumen. Using rings with holes (Figure 5A) or thin structures that are woven through the cables (Figure 5B) are, therefore, undesired. After careful consideration, it was chosen to use external ring magnets to prevent radial movement of the cables. Ring magnets that surround the cables, will attract the cables and thus create a tubular structure and lumen without protruding structures at the inside, providing that the cables are made of a ferromagnetic material, such as steel (Figure 5C).

The ring magnets must be located at a certain distance from each other. A large distance will increase the risk of collapse of the cable structure during bending of the tube. There is, however, a trade-off, as more magnets will increase the magnetic normal force acting on the cables, increasing the friction between the cables and the ring magnets, which in turn will increase the force needed to translate the cables. The maximum distance between the ring magnets that would still avoid lumen collapse even in a curved position was determined empirically. To keep the magnets at a certain distance while also allowing flexibility, we decided to use compression springs as distance holders. The entire shaft was covered by a heat shrink tube in order to prevent tissue escaping from the shaft during transportation.

### Actuation

The handle must allow for easy actuation of the transport mechanism and thus the cables. It was decided to design the handle such that the system can be actuated manually, as this limits the number of required parts for this prototype. In a future version, the use of motorised actuation might be desired as it could result in faster and a more constant transport rate. In the study of Sakes et al. (2020), reliable tissue transport was achieved with the sequence of five blades moving in the desired transport direction, with the remaining 6th blade moving in the opposite direction (5:1). Following this design, it was chosen to divide the eighteen cables in six groups of three cables each.

**FIGURE 6:**
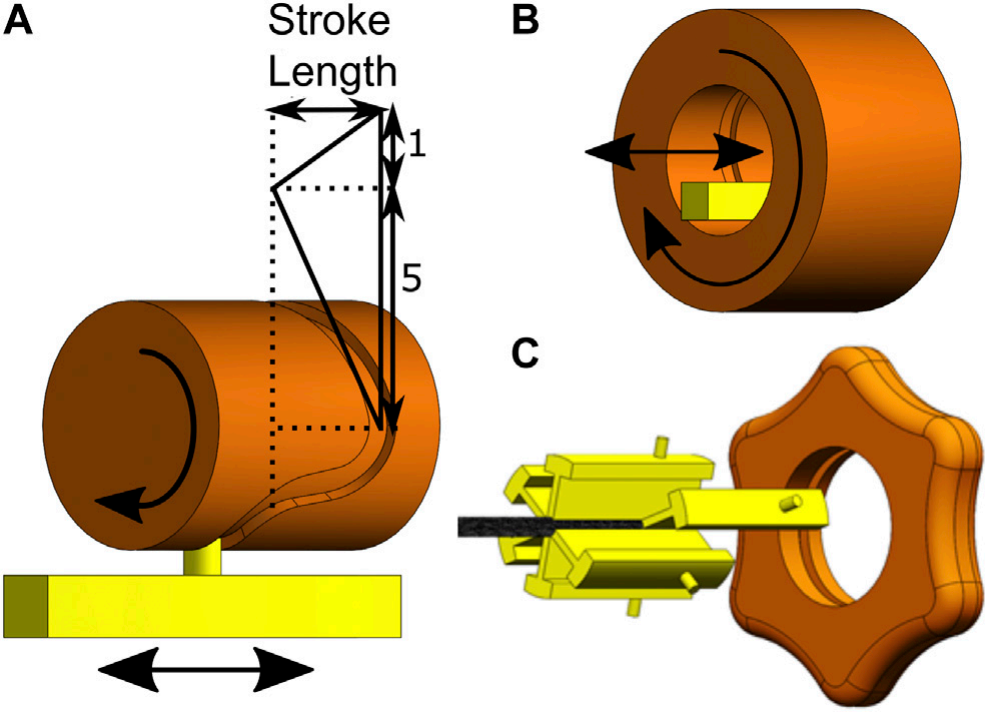
Representation of the cam design. **(A)** The effect of the groove on the motion sequence of the sliders. **(B)** Schematic drawing of the inside-out barrel cam. **(C)** The cam (orange) and sliders (yellow) of the transport mechanism.

Each cable group is glued to an aluminium slider, such that the entire cable group can be actuated simultaneously by translating the slider. In the previously developed prototype by Sakes et al. (2020), a barrel cam, consisting of a cylindrical part with a groove in which the pin of a slider fits, was used to actuate the blades, see Figure 6A. The groove was designed in such a way that five sliders are moving in the desired direction while one slider moves in the opposite direction, resulting in the 5:1 sequence with a stroke length of 5.2 mm. A similar design was used for cable actuation in the flexible prototype, with one major difference: in the flexible prototype, translational movements of the sliders in the desired sequence (5:1) are achieved by a new inside-out barrel cam design. The inside-out barrel cam is hollow and surrounds the sliders, allowing the tissue to be transported through the cam and handle such that it can easily be removed, see Figure 6B. In the prototype, the outside of the barrel cam has a star-shaped knob to allow for precise manual actuation of the transport mechanism, see Figure 6C. Furthermore, a crank was connected to the cam to allow for faster and easier actuation of the cam. Depending on the clockwise or counter clockwise rotation of the cam, the transport direction will reverse, making the same system usable to transport substances from and to the target location.

### Prototype

**FIGURE 7:**
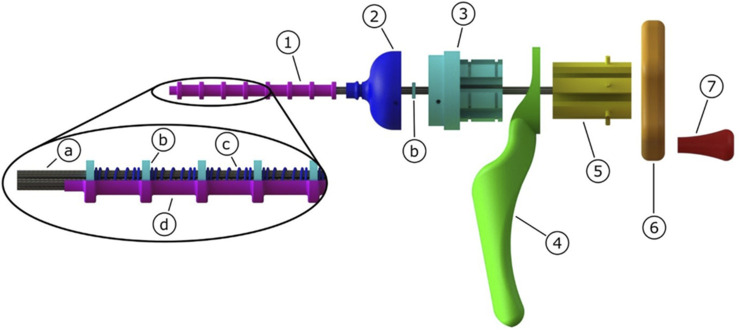
Schematic representation of the flexible transport mechanism consisting of 1) flexible shaft, 2) tip, 3) slider house, 4) hand grip, 5) sliders, 6) cam, 7) crank. The flexible shaft consists of a) cables that form a lumen due to their attraction to the b) ring magnets. The ring magnets are kept in place by c) compression springs, and all is enclosed by a d) heat shrink tube.

Figure 7 shows the final prototype. The flexible tissue transport mechanism consists of eighteen galvanised steel cables (Engelmann, 1 × 7, Ø 0.6 mm) arranged in six groups of three cables, that form a tubular structure due to the eight neodymium ring magnets (Conrad Components Permanent magnet Ring N35M, OD 10 mm, ID 5 mm, length 2 mm) that are placed around the cables. In between two ring magnets, a compression spring (ID 6.5 mm, Ø wire 0.6 mm, free length 12 mm) is placed. The shaft with a length of 115 mm was partly covered by a heat shrink tube (Ø 9.5 mm) leaving 15 mm of cables uncovered at the tip. The cables can translate in the longitudinal direction by sliding through the ring magnets. Three cables are glued to each of the sliders (manufactured using electrical discharge machining). These three cables will thus be simultaneously actuated by the inside-out cam (manufactured using a Perfactory 4 Standard 3D-printer by EnvisionTec).

**FIGURE 8:**
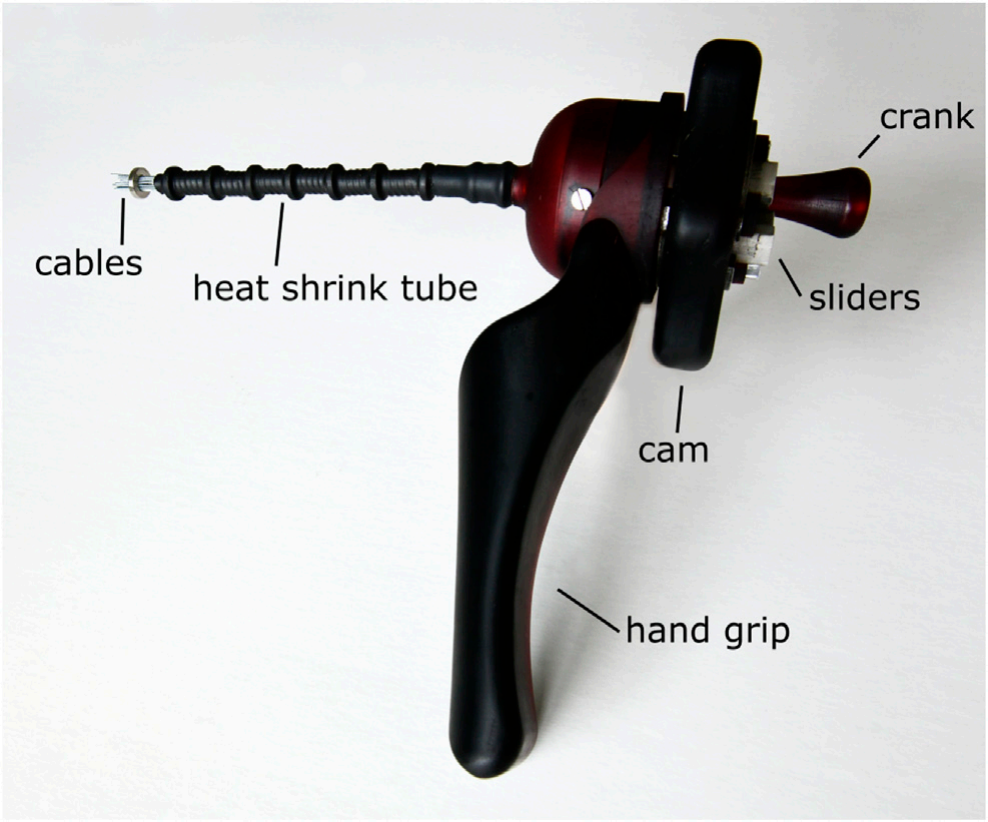
Photo of the flexible ovipositor-inspired tissue transport mechanism with an extra ring magnet around the tip of the shaft to elucidate the working principle.

**FIGURE 9:**
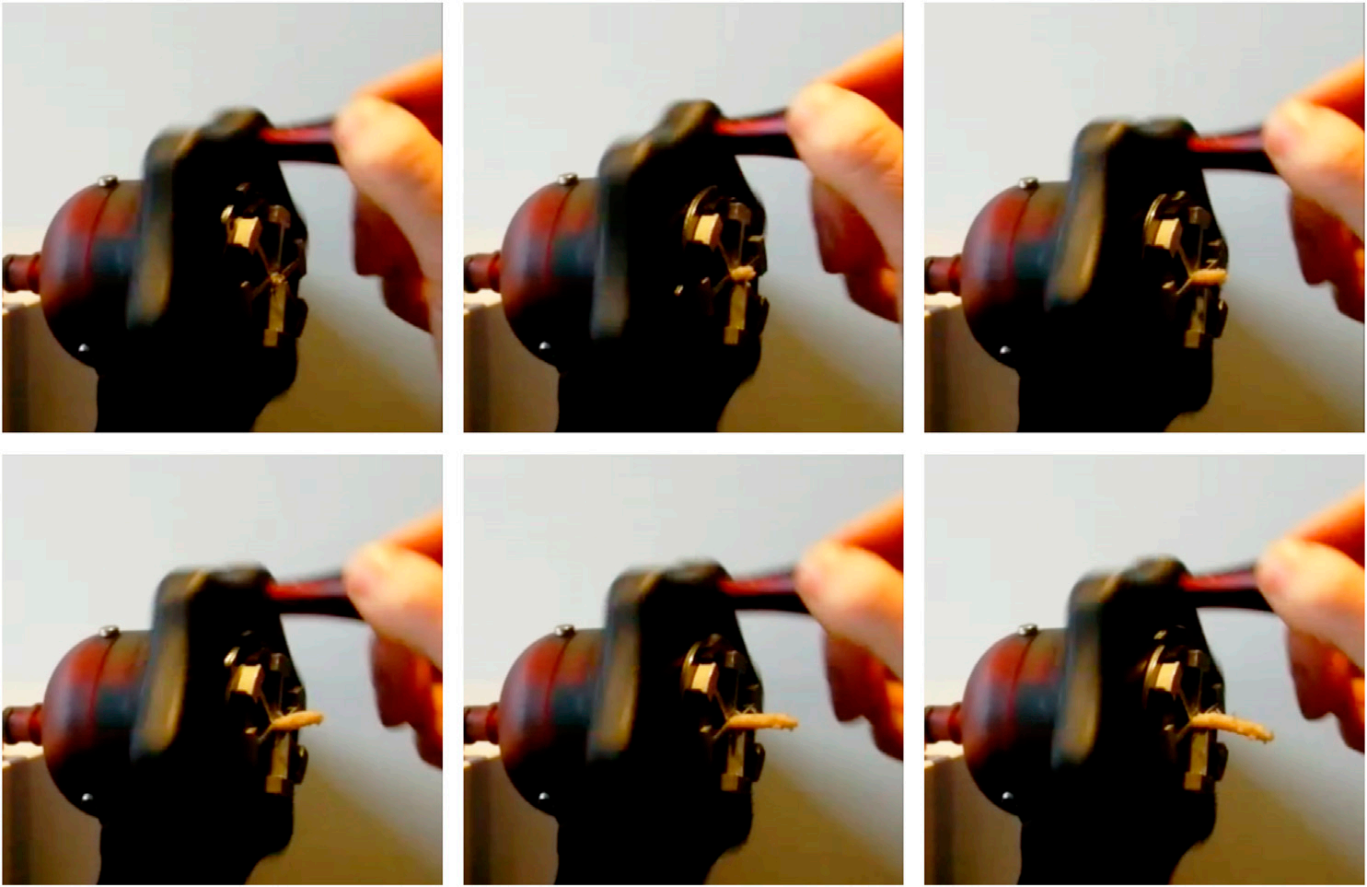
Photo of the tissue leaving at the end of the tissue transport mechanism using manual actuation. The photos were taken one actuation cycle apart.

A photo of the prototype is shown in Figure 8. After the fabrication and assembly of the prototype, the transport mechanism was tested. Tissue phantoms were placed inside the lumen at the tip of the transport mechanism. By manually actuating the system, the phantoms were transported through the flexible shaft and the handle. Figure 9 shows one of the tissue phantoms exiting the handle after being transported through the mechanism. A video showing the working principle of the flexible wasp-inspired tissue transport mechanism can be found in the Supplementary Material.

## Proof-of-Principle Experiment

### Experiment Goal

The performance of the transport mechanism was evaluated during a proof-of-principle experiment. The goal of this proof-of-principle experiment was threefold: 1) to determine the effect of the shaft curvature on the performance of the friction-based transport mechanism, 2) to evaluate the effect of shaft orientation (horizontal vs vertical) on the transport performance, and 3) to determine the effect of the rotational velocity of the cam on the transport performance.

### Experimental Facility

#### Experiment Set-Up

**FIGURE 10:**
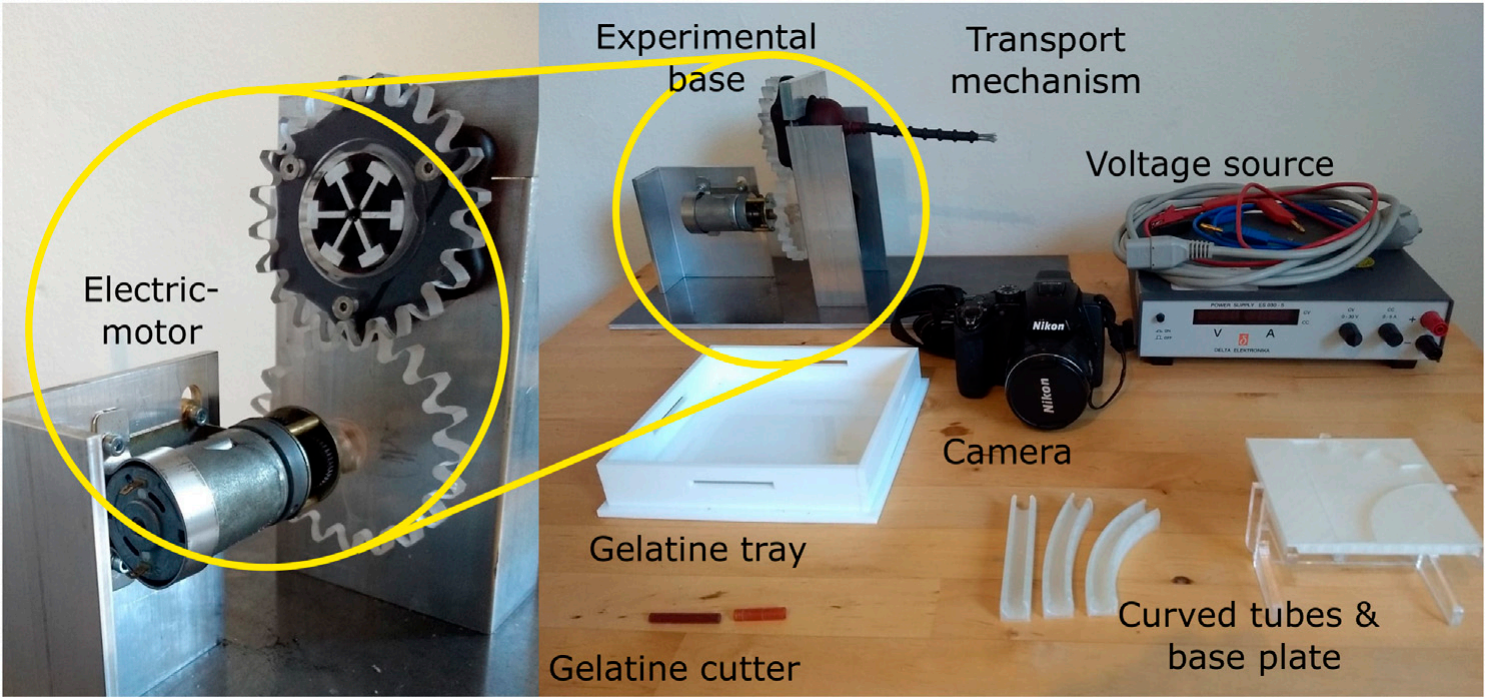
Experimental facility. The experimental facility consisted of an electromotor connected to a voltage source and the handle of the transport mechanism through a gear mechanism, the transport mechanisms placed inside an aluminium frame, a camera to record the experiments, a based plate containing 3D-printed tubes for the shaft curvature experiment, and a gelatine tray with cutter for the manufacturing of the tissue phantoms.

Figure 10 shows the experimental set-up. The transport mechanism was initially designed as a handheld manually-actuated prototype. However, for testing purposes, an electric motor (Igarashi 33GN2738-132-GV-5 12.0V with a 75:1 gearbox) and a voltage source were connected to the mechanism, such that the rotational velocity of the cam could be precisely controlled and adjusted. The transport mechanism was mounted in an aluminium experimental base to ensure correct shaft orientation. When testing the transport mechanism in the vertical position, the standard was rotated 90^o^, such that the shaft was in vertical orientation. 3D-printed straight and curved tubes that could be placed in a base plate were used to ensure correct curvature of the shaft. A camera (Nikon Coolpix P500) was used to film the experiment and to later identify the number of cam rotations needed to transport the gelatine through the transport mechanism.

#### Gelatine Tissue Phantoms

The transport performance was tested by transporting gelatine tissue phantoms. Gelatine is a widely used substance for soft tissue phantoms, as it is easy to handle and can produce phantoms with similar Young’s moduli as real tissue (Cournane et al., 2012; Leibinger et al., 2016). A phantom with 10 wt% of dry gelatine powder results in a phantom with a Young’s modulus around 70 KPa (Karimi and Navidbakhsh, 2014). This corresponds to the Young’s modulus of tendons (Arda et al., 2011), glandular breast tissue and prostate tissue (Krouskop et al., 1998). The gelatine tissue phantoms were made by mixing dry gelatine powder (dr. Oetker) with tap water of approximately 90°C. The mixture was stirred until the gelatine was completely dissolved. Subsequently, the gelatine mixture was poured into a gelatine tray (see Figure 10) to create a gelatine layer with a thickness of 20 mm and was left to cool for 24 h at approximately 7°C. The gelatine tray was removed from the fridge an hour before the experiments, to allow the tissue samples to reach room temperature. A 3D-printed gelatine cutter was used to cut gelatine cylinders with a 5 mm diameter and 20 mm height. The cutter was, subsequently, used to place the tissue sample into the lumen of the transport mechanism.

### Experiment Variables

#### Independent Variables

The following variables were manipulated during the experiment:

• Shaft curvature: The shaft was tested in straight position, as well as in two circular curves: Curve 1 with an angle of 30^o^ over a length of 8 cm and Curve 2 with an angle of 60^o^ over a length of 8 cm.
• Shaft orientation: During MIS, the transport mechanism will be used in different orientations relative to gravity. The transport mechanism was therefore tested in two extreme scenarios: 1) in a horizontal orientation in which the gravity is perpendicular to the transport direction and 2) in a vertical orientation in which gravity is opposite to the transport direction.
• Rotational velocity: To determine the effect of the rotational velocity of the cam on the transport performance, the experiments were carried out with three rotational velocities: 25, 53, and 80 RPM.

#### Dependent Variables

The following variables were measured during the experiment:

• Transport rate: Visual analysis of video recordings were used to determine the transport time ${\text{t}}_{\text{transport}}\;$ [s]. The transport rate [mm/s] was calculated by subdividing the distance over which the tissue phantom was transported $d_{\text{transport}}$ [mm] by the transport time $t_{\text{transport}}$ [s] needed to achieve this, see Eq. 4. The transport distance $d_{\text{transport}}$ is 163 mm, which is equal to the length of the shaft surrounded by heat shrink tube plus the length of the handle, minus the length of the tissue phantom.

$$\text{transport}\;\text{rate}=\frac{d_{\text{transport}}}{t_{\text{transport}}}$$ (4)

• Stroke efficiency: In order to determine the efficiency of the tissue transport, the amount of slip between the cables and the tissue phantom was measured. The stroke efficiency $\eta_{\text{stroke}}\;$ [%] was calculated by dividing the measured transport distance per stroke $d_{\text{measured}}$ [mm/stroke], by the theoretical maximum transport distance per stroke $d_{\text{theoretical}}$ [mm/stroke], multiplied by 100%, see Eq. 5. The measured transport distance per stroke $d_{\text{measured}}$ can be found by dividing the total transport distance $d_{\text{transport}}$ [mm] by the required number of strokes, $n_{\text{strokes}}$ [−], which is equal to the number of cam rotations. The number of cam rotations was determined by visual analysis of the video recordings. The theoretical maximum transport distance $d_{\text{theoretical}}$ depends on the motion sequence (5:1) and the stroke length of the sliders (5.2 mm). The theoretical transport distance per stroke is equal to $\frac{6}{5}\times 5.2\;\text{mm}=6.24\;\text{mm}$ in this prototype.

$$\text{stroke}\;\text{efficiancy}=\frac{d_{\text{measured}}}{d_{\text{theoretical}}}\cdot 100\%$$ (5)

### Experiment Protocol

The proof-of-principle experiment was divided in three sub-experiments.

**FIGURE 11:**
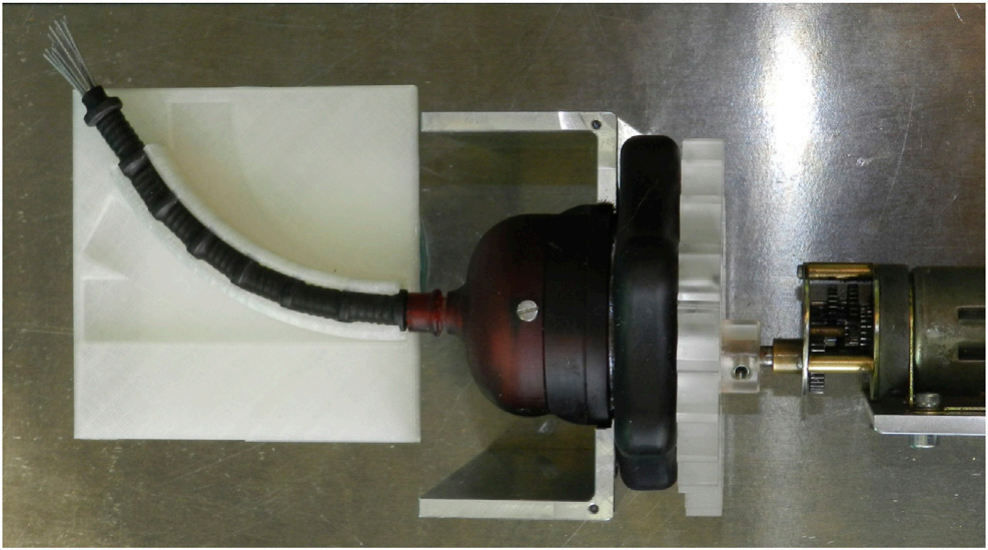
Photo showing the flexible shaft of the transport mechanism placed inside the 30° curved tube.

I. Curvature Test: In order to test the effect of the shaft curve on the transport performance, the mechanism was tested with the shaft in the straight position and in the two curved positions. Figure 11 shows the flexible shaft in the curved position.
II. Orientation Test: In order to test the effect of the shaft orientation on the transport performance, the mechanism was tested in the horizontal and the vertical orientation with the transport direction opposing gravity.
III. Rotational Velocity Test: In order to test the effect of the rotational velocity on the transport performance, both the Curvature Test and the Orientation Test were performed at the three rotational velocities.

Each test condition was repeated six times. The tests started with placing the gelatine tissue phantoms inside the lumen at the tip of the transport mechanism. In order to ensure proper contact between the cables and the gelatine, the gelatine was transported manually by rotating the cam until the gelatine sample was entirely inside the lumen surrounded by the heat shrinking tube. The test ended once the tip of the gelatine cylinder reached the proximal end of the transport mechanism, which was determined by eye. This way the gelatine cylinder was completely surrounded by cables for the duration of the test.

### Data Analysis

For each condition, the mean and the standard deviation were determined for both the transport rate and the stroke efficiency. The statistical analysis was conducted by performing ANOVA analyses and t-tests on the data. All data analysis was performed with MATLAB R2019B.

## Results Proof-of-Principle Experiment

### Curvature Test

**FIGURE 12:**
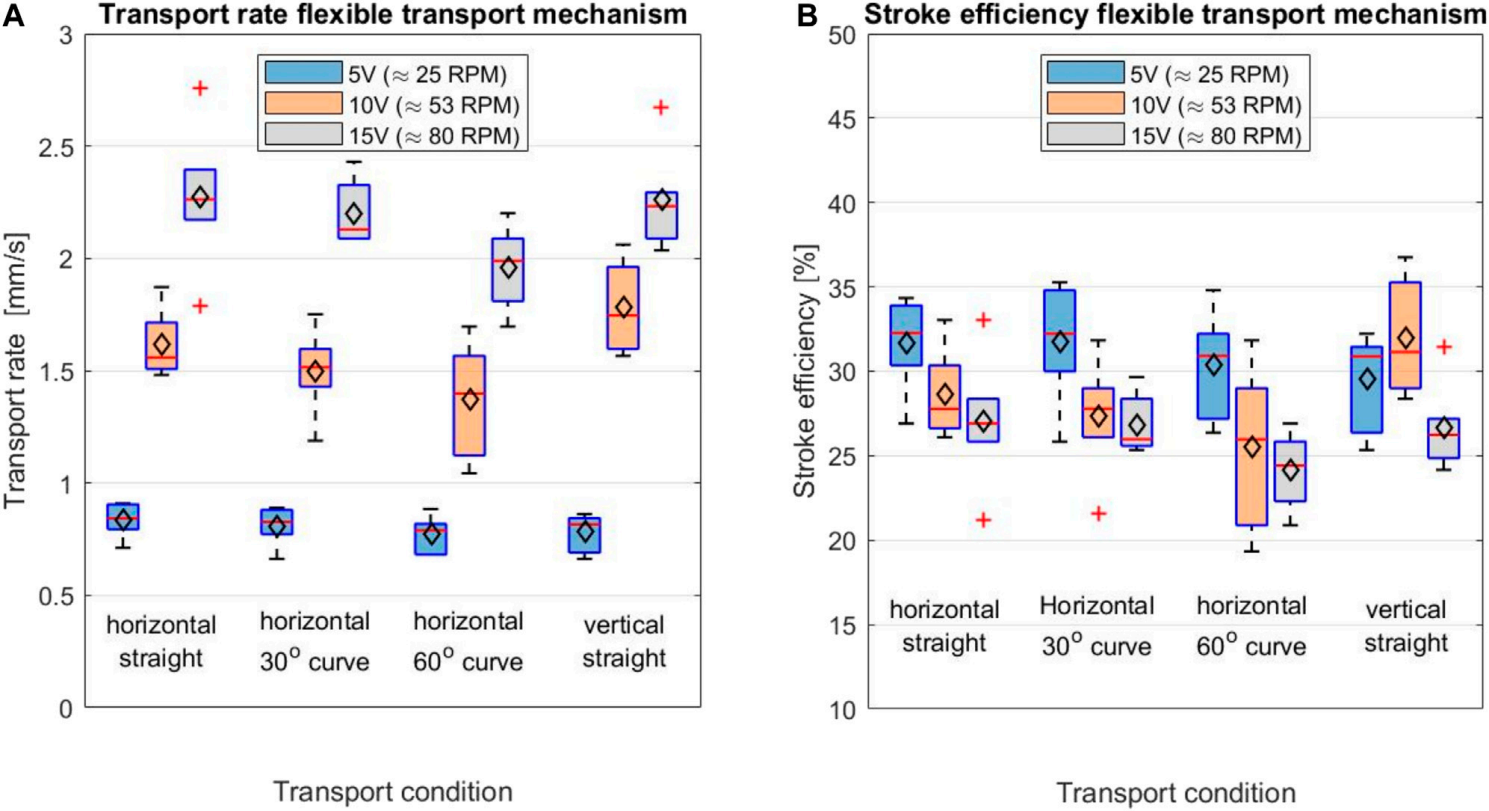
Boxplot displaying **(A)** the transport rate [mm/s] and **(B)** the stroke efficiency [%] for different test conditions; the shaft in horizontal orientation while being straight, in 30° and 60° curved position and with the shaft in vertical orientation. The maximum and minimum values are indicated by the outermost horizontal solid lines on each of the boxplots. The median is indicated by the horizontal red line in each boxplot, while the mean is indicated by the black diamond.

The boxplots in Figure 12 summarise the results of the experiment. The mean transport rate for the shaft in the straight, 30^o^ curved and 60^o^ curved position can be found in Table 1. Transport rates of 0.83 ± 0.08, 0.81 ± 0.09, and 0.77 ± 0.08 mm/s were found for the straight, 30^o^ curved and 60^o^ curved positions at a rotational velocity of 25 RPM, respectively. For a rotational velocity of 53 RPM, higher transport rates of 1.61 ± 0.15, 1.50 ± 0.19, and 1.37 ± 0.25 mm/s for the straight, 30^o^ curved and 60^o^ curved position were found, respectively. Finally, as expected, the highest transport rates of 2.27 ± 0.32, 2.20 ± 0.14, and 1.96 ± 0.19 mm/s for the straight, 30^o^ curved and 60^o^ curved position were found for a rotational velocity of 80 RPM, respectively. There was no statistical effect of the shaft curvature on the transport rate.

**TABLE 1:** Overview of the transport rate [mm/s] of the friction-based transport mechanism for different shaft orientations, curvatures and rotational velocities.

Transport rate [mm/s]	Test condition			
Rotational velocity	Straight horizontal	30° curved	60° curved	Straight vertical
25 RPM	0.83 ± 0.08	0.81 ± 0.09	0.77 ± 0.08	0.78 ± 0.08
53 RPM	1.61 ± 0.15	1.50 ± 0.19	1.37 ± 0.25	1.78 ± 0.20
80 RPM	2.27 ± 0.32	2.20 ± 0.14	1.96 ± 0.19	2.26 ± 0.23

### Orientation Test

The effect of the shaft orientation of the transport mechanism on the transport rate was investigated by performing a two-tailed t-test. This showed that there was no statistical effect of shaft orientation on the transport rate at the 25, 53 and 80 RPM rotational velocities of the cam (*p* = 0.284, *p* = 0.143, and *p* = 0.928, respectively).

### Rotational Velocity Test

Based on the one-way ANOVA, a statistically significant difference was found in the transport rate at different rotational velocities of the cam. The average stroke efficiency for the test conditions: straight horizontal, 30^o^ curved, 60^o^ curved and vertical straight, can be found in Table 2. The one-way ANOVA test showed that there is a statistically significant effect of the rotational velocity of the cam on the stroke efficiency for the horizontal 30^o^ curved (*p* = 2.39·10^−2^), horizontal 60^o^ curved (*p* = 1.99·10^−2^) and the vertical straight condition (*p* = 2.89·10^−2^). There was no statistical significance found on the rotational velocity on the stroke efficiency in the horizontal straight transport condition (*p* = 6.17·10^−2^).

**TABLE 2:** Overview of the stroke efficiency [%] of the friction-based transport mechanism for different shaft orientations, curvatures and rotational velocities.

Stroke efficiency [%]	Test condition			
Rotational velocity	Straight horizontal	30° curved	60° curved	Straight vertical
25 RPM	32 ± 3	32 ± 3	30 ± 3	30 ± 3
53 RPM	29 ± 3	27 ± 3	25 ± 5	32 ± 4
80 RPM	27 ± 4	27 ± 2	24 ± 2	27 ± 3

## Discussion

### Main Findings

The proof-of-principle experiments show that the shaft curvature and shaft orientation (horizontal vs vertical) do not influence the transport rate significantly. The rotational velocity of the cam did have a significant effect on the transport rate of the tissue. The transport rates for the shaft in straight position were 0.83 ± 0.08 mm/s, 1.61 ± 0.15 mm/s and 2.27 ± 0.32 mm/s with a rotational velocity of the cam of 25, 53 and 80 RPM, respectively. An increase of the rotational velocity of the cam resulted in an increase of the transport rate. The prototype has been designed for manual actuation, but in future designs motorised actuation might be desirable to further increase the transport rate. Although increasing the rotational velocity of the cam resulted in an increase of the tissue transport rate, the stroke efficiency showed a decrease when increasing the rotational cam velocity in the horizontal test conditions. This decrease in stroke efficiency could indicate that there is a limit to the tissue transport rate that can be achieved by increasing the rotational velocity of the cam.

The rigid tissue transport mechanism, which also uses friction-based transport, has a comparable transport rate of 1.49 mm/s when used with similar circumstances (9wt% gelatine tissue phantoms, rotational velocity cam = 46 RPM) (Sakes et al., 2020). This transport rate is 50–165 times lower than the clinically used morcellators that use suction-based transport. However, during interventions that are performed in close proximity to delicate structures and in which the transport rate is of secondary interest, the use of friction-based transport could still be beneficial (Sakes et al., 2020).

One of the main disadvantages of the currently used suction-based instruments is that miniaturisation is challenging as this would require a larger pressure difference than what is achievable. This problem does not arise when miniaturising the proposed friction-based transport mechanism. In the current design, miniaturisation of the outer diameter of the shaft is limited by the size of the used ring magnets. Using smaller ring magnets could allow for a smaller outer diameter, however, the magnetic force acting on the cables is dependent on the volume of the magnet. This means that at some point the magnets will not be able to generate the required magnetic force to ensure the lumen formation of the cables. Down scaling of the cables would lower the required magnetic force and could thus allow for further miniaturisation of the prototype. Besides this, alternative methods to form a lumen of the cables could be investigated. For instance, braiding the cables to the springs with a thin and smooth wire. These methods might be more space-efficient and could therefore be beneficial for minimally invasive surgery.

Elongation of the flexible shaft would increase the number of magnets required to ensure the open lumen over the entire length of the shaft. This could pose a challenge, as more magnets will increase the friction force between the cables and the magnets during the translating motion of the cables. Research is needed to get more insight in the effects of elongating the shaft.

### Limitations and Future Research

The performance of the transport mechanism was tested by using gelatine tissue phantoms. Although gelatine is commonly used as a tissue phantom, the homogeneous structure of gelatine could influence the transport rate. Furthermore, in the research of Sakes et al. (2020), it was found that there is a statistically significant effect of the gelatine density and thus the tissue elasticity on the transport rate. Therefore, it would be valuable to repeat these tests with real tissue in clinical *ex-vivo* and *in-vivo* settings. *In-vivo* testing would require the prototype to be biocompatible hence, alternatives must be found for the materials now used in the prototype, such as the galvanised cables and the heat shrink tube. Furthermore, as this transport mechanism is expected to be used in combination with a tissue separating instrument, it would be valuable to test this transport mechanism in combination with such an instrument or look into the possibilities of adding a tissue separating grasper at the tip of the instrument.

The ovipositor on which the flexible transport mechanism is based is steerable. With slight adjustments, the prototype could also be made steerable. For instance, by adding steering cables that are connected to the tip of the flexible shaft and run along the shaft. Pulling on one of these cables would result in bending of the flexible shaft allowing the surgeon to actively steer the tip of the transport mechanism to the target location. Similar principles have been used before to create steerable catheters (Ali et al., 2019), endoscopes (Breedveld et al., 2005) and laparoscopic instruments (Jelínek et al., 2014).

The proposed prototype shows that friction-based transport can be used in a flexible system which allows the flexible transport mechanism to transport tissue without the adverse effects, such as clogging, that are linked to suction-based instruments. In the future, this design might serve as an alternative for the currently used flexible suction-based instruments and can be used in a wide variety of minimally invasive interventions.

## Conclusion

This paper presents the design of a novel flexible tissue transport mechanism that uses a wasp-ovipositor-inspired transport method. This method allows for continuous tissue transport while eliminating clogging as a sub-optimal behaviour mode of the currently used suction-based transportation mechanisms. The prototype could transport 10wt% gelatine tissue phantoms with no significant difference depending on the shaft curvature and orientation. The transport performance of the flexible transport mechanism is promising and could in future be used in a wide variety of medical interventions, such as tissue removal during minimally invasive procedures.

## Data Availability

The raw data supporting the conclusion of this article will be made available by the authors, without undue reservation.
